# Positive to negative zero-field cooled exchange bias in La_0.5_Sr_0.5_Mn_0.8_Co_0.2_O_3_ ceramics

**DOI:** 10.1038/srep25703

**Published:** 2016-05-11

**Authors:** Cui Shang, Shaopu Guo, Ruilong Wang, Zhigang Sun, Haibo Xiao, Lingfang Xu, Changping Yang, Zhengcai Xia

**Affiliations:** 1 Wuhan National High Magnetic Field Center & School of Physics, Huazhong University of Science and Technology, Wuhan 430074, People’s Republic of China; 2 Hubei Collaborative Innovation Center for Advanced Organic Chemical Materials, Hubei Key Laboratory of Ferro & Piezoelectric Materials and Devices, Faculty of Physics and Electronic Science, Hubei University, Wuhan 430062, People’s Republic of China; 3 State Key Lab of Advanced Technology for Materials Synthesis and Processing, Wuhan University of Technology, Wuhan 430070, People’s Republic of China

## Abstract

Exchange bias effect obtained after zero-field cooling from unmagnetized state usually exhibits a shift of hysteresis loop negative to the direction of the initial magnetic field, known as negative zero-field cooled exchange bias. Here, positive zero-field cooled exchange bias is reported in La_0.5_Sr_0.5_Mn_0.8_Co_0.2_O_3_ ceramics. In addition, a transition from positive to negative exchange bias has been observed with increasing initial magnetization field and measurement temperature. Based on a simple spin bidomain model with variable interface, two type of interfacial spin configuration formed during the initial magnetization process are proposed to interpret the observed phenomenon.

Since first reported in Co/CoO system by Meikle John and Bean^1^, exchange bias (EB) effect has received considerable attention due to its importance in fundamental research and memory devices applications like random access magnetic storage units and spin valves^2^,^3^,^4^,^5^. It manifests itself by a shift of the hysteresis loop away from the magnetic field axis, and the magnitude of this shift is referred to as EB field, *H*_EB_. Although the origin of EB is still not fully understood, it is generally accepted that it is related to the interfacial ferromagnetic (FM)-antiferomagetic (AFM) exchange coupling interaction across the interface and the AFM unidirectional anisotropy hold a key role to decide the magnitude and sign of the shift^6^,^7^,^8^,^9^. Conventionally, AFM unidirectional anisotropy is induced from an imprint of the FM unidirectional anisotropy during a field-cooling (FC) process through the Néel temperature (*T*_N_) of AFM subsystem, and the FM coupling results in a shift of the hysteresis loop in the opposite (‘negative’) direction to the cooling field, defined as negative EB. Positive EB effect can be observed upon strong cooling field in some cases, in which the interfacial exchange interaction is believed to be AFM^10^,^11^. In the measurement process of obtaining both negative and positive EB effect, the FM-AFM interface is supposed to be fixed due to sufficiently large AFM anisotropy energy. Hence, it is high unexpected to observe zero-field cooled EB (ZFC-EB) effect, in another word, a shift of the magnetic hysteresis loop center away from zero field after zero-field cooling from unmagnetized state due to the formation of two oppositely oriented AFM domains.

Though ZFC-EB effect with small *H*_EB_ was reported in bilayer systems early^12^, it receives wide acceptance until the observation of large ZFC-EB effect with *H*_EB_ up to 1300 Oe in Ni_50_Mn_37_In_13_ alloy by wang *et al*.^13^, in which the magnitude of the shift can be tuned by the strength of initial magnetization field. From then on, the ZFC-EB effect has been observed in different systems, such as Mn_2_PtGa alloy^14^, BiFeO_3_–Bi_2_Fe_4_O_9_ nanocomposite^15^, YMnO_3_ nanoparticles^16^, Ni_2_Mn_1.4_Ga_0.6_ alloy^17^, La_1.5_Sr_0.5_CoMnO_6_ polycrystalline ceramics^18^, Pr_1−*x*_Ca_*x*_MnO_3_ nanosheet^19^, Ni_50_Mn_36_Co_4_Sn_10_ alloy^20^, antiperovskite compound PdNCr_3_^21^, Co_0.8_Cu_0.2_Cr_2_O_4_^22^ and Y_0.9_Pr_0.1_CrO_3_ ceramics^23^. In most cases, the appearance of such ZFC-EB effect is related to newly established interface with AFM unidirectional anisotropy, and the formation or growth of FM (or superferromagnet, SFM) domains at the expense of pinning subsystem (AFM matrix or spin glass) has been proposed as a possible way^13^,^16^,^17^,^18^,^19^,^20^,^21^. However, almost all these studies show only the negative ZFC-EB effect, in another word, shift of the hysteresis loop in the opposite direction to the initial magnetization field. It is highly desired of obtaining tunable positive and negative ZFC-EB systematically for an intensively understanding of the EB origination. In present work, we report a transition from positive to negative ZFC-EB effect by increasing initial magnetization field and measurement temperature in La_0.5_Sr_0.5_Mn_0.8_Co_0.2_O_3_ ceramics, which can be ascribed to the evolution of the interfacial spin configuration during the initial magnetization process.

## Results

**Figure 1:**
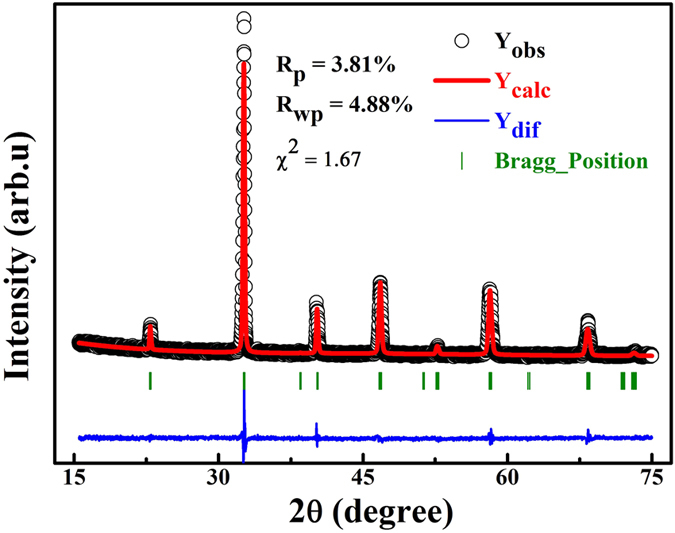
Room temperature XRD and refinement result of sample La_0.5_Sr_0.5_Mn_0.8_Co_0.2_O_3_. The observed and calculated patterns are denoted by circle and solid line, respectively. The blue solid line at the bottom of the panel shows the difference of the observed and calculated patterns. Green bars correspond to Bragg positions.

Figure 1 shows the room temperature XRD patterns of La_0.5_Sr_0.5_Mn_0.8_Co_0.2_O_3_, in which no trace of any impurity phases is discernible. The refined data obtained with the Rietveld refinement program FULLPROF are also shown in Fig. 1. The best fitting of the observed diffraction peaks indicates the sample with tetragonal structure and the space group *I4/mcm*. The refined lattice parameters (*a, b* and *c*) and unit volume (*V*) of La_0.5_Sr_0.5_Mn_0.8_Co_0.2_O_3_ are *a* = *b* = 5.4475 (5) Å, *c* = 7.7208 (9) Å, and *V* = 229.12 (4) Å^3^.

**Figure 2:**
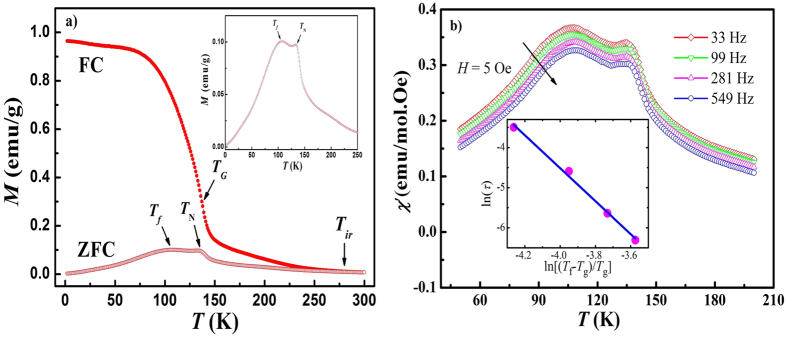
(**a**) Temperature dependence of magnetization *M*(*T*) measured at 50 Oe after FC and ZFC procedures. The inset shows the enlarge scale of ZFC curve. (**b**) Temperature dependence of the real part *χ*′(*T*) of ac susceptibility measured at different frequencies with an ac magnetic field of 5 Oe. The inset shows the fit using the power law.

Figure 2(a) shows the temperature dependence of magnetization *M*(*T*) measured at 50 Oe with FC and ZFC procedures. The ZFC and FC magnetization curves start to separate from each other at an irreversibility temperature around *T*_*ir*_ ~ 280 K, and they begin to diverge strongly at low temperatures, which is a strongly pronounced typical feature of both classical spin glasses (SG) and phase-separated manganite systems with coexisting FM clusters and an AFM matrix. Two successive peaks at 133 K and 106 K, recognized as *T*_N_ and freezing temperature *T*_*f*_ respectively, can be observed in the ZFC curve (see inset). As reported before, in canonical SG systems *T*_*ir*_ is very close to *T*_*f*_ typically^24^. However, *T*_*ir*_ is far above *T*_*f*_ in present case, indicating the possibility of a cluster glass (CG) state at low temperatures^25^,^26^. Therefore, the rapid rise of magnetization around the temperature *T*_*G*_ (137 K for FC and 140 K for ZFC, determined from the minimum of *dM*/*dT* vs *T* curve) could be associated with the occurrence of a short range FM ordering, forming FM clusters. Additionally, *T*_*f*_ could be considered as the freezing temperature of the FM clusters.

In order to get further insight into the magnetic properties of this system, ac susceptibility under different frequencies was measured. Figure 2(b) shows the temperature dependence of the real part *χ*′(*T*) of ac susceptibility measured at different frequencies with an ac magnetic field of 5 Oe. Two pronounced peaks, being close to *T*_*N*_ and *T*_*f*_ respectively, can be observed. It is remarkable that the position of *T*_*f*_ is frequency dependent and obviously shifts toward a higher temperature with increasing frequency. Such behavior indicates a glassy character of the sample. The critical slowing-down power law, which assumes a true equilibrium phase transition with a divergence of relaxation time near the freezing point, was employed to analyze spin dynamics of the glass-like state. According to the power law, the temperature dependence of τ could be described by 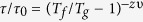 28, where *T*_*f*_ is the peak temperature measured at frequency *f, T*_*g*_ is the critical temperature for SG ordering which is equivalent to *T*_*f*_ as *f* → 0, *zν* is a constant exponent, and *τ*_0_ is the characteristic time scale for spin dynamics (the shortest relaxation time of the system). The best fit shown in the inset of Fig. 2(b) gives the value of *T*_*g*_ = 106.5 K (nearly equal to the dc value found from dc magnetization), *zν* = 4.11, *τ*_0_ = 7.92 × 10^−10^ s. The long spin flipping time *τ*_0_ (~10^−12^ − ~10^−14^ for classical SG compounds) and relative small critical exponent *zν* suggests a CG state nature at low temperature magnetism in the La_0.5_Sr_0.5_Mn_0.8_Co_0.2_O_3_ ceramics^27^,^28^, in another word, the state of FM clusters embodied in AFM matrix.

**Figure 3:**
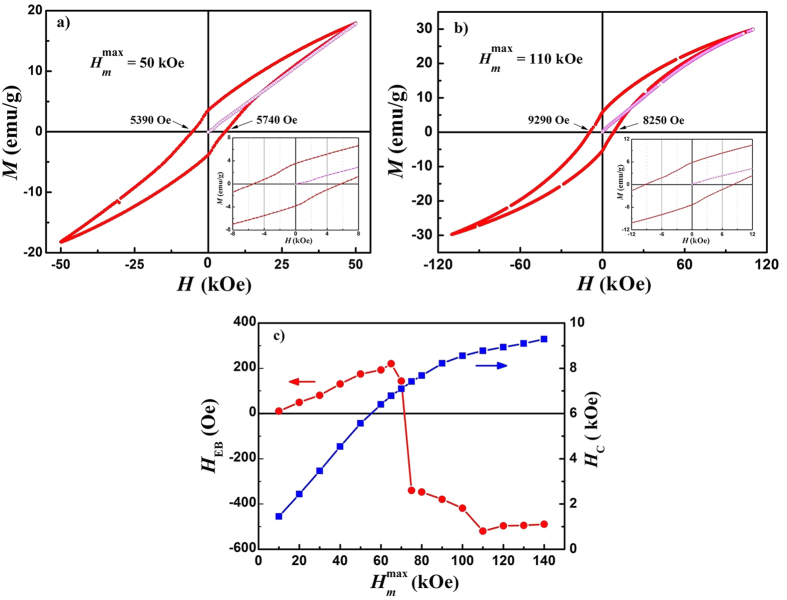
*M* (*H*) loops at 4.2 K within full scale of magnetic field with 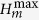 = 50 kOe (**a**) and 110 kOe (**b**). The insets are the enlarge scale at low field; (**c**) *H*_EB_ and *H*_C_ as a function of 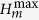 at 4.2 K after ZFC.

The *M*(*H*) loops of La_0.5_Sr_0.5_Mn_0.8_Co_0.2_O_3_ ceramics measured at 4.2 K with various magnitudes of the initial magnetization field, i.e. maximum measurement field (different 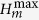) at 4.2 K after ZFC from 300 K. The unmagnetized initial state at low temperatures can be ensured as 300 K is above the *T*_*G*_. As representatives, the *M*(*H*) loops with 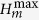 = 50 and 110 kOe are shown in Fig. 3(a,b) respectively. Magnetization at the starting point of the initial magnetization curve (open circles) is close to zero, indicating that the initial state at 4.2 K is an unmagnetized state. Obvious positive and negative shift of the hysteresis loop along the magnetic field axis, recognized as positive and negative ZFC-EB effect, can be observed for 50 and 110 kOe, respectively. Figure 3(c) shows *H*_EB_ and coercivity (*H*_C_) as a function of 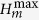. The *H*_EB_ and *H*_C_ were calculated using *H*_EB_ = (*H*_1_ + *H*_2_)/2 and *H*_C_ = |*H*_1_ − *H*_2_|/2, where *H*_1_ and *H*_2_ are the left and right fields at which the magnetization becomes zero. We can find that *H*_EB_ exhibits positive value at low field, and first increases with the increase of 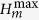, reaching a maximum value of 220 Oe at 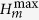 = 65 kOe and then decreases. Further increasing 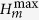, *H*_EB_ would change to negative value, reaching a minimum value of −520 Oe at 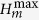 = 110 kOe and then increases slowly. The phenomenon of positive ZFC-EB effect and its transition to negative ZFC-EB at high initial magnetization field indicate that the value and sign of *H*_EB_ are strongly dependent on the magnitude of the initial magnetization field.

**Figure 4:**
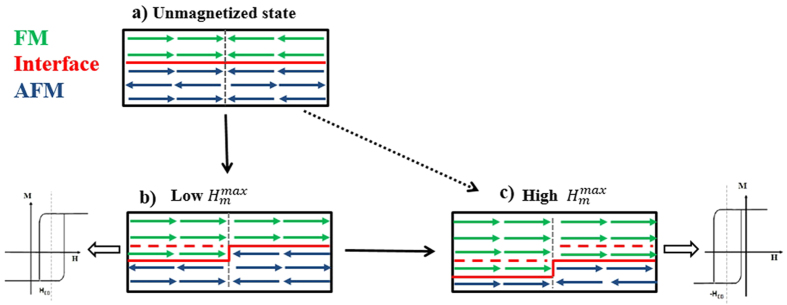
Schematic diagrams of the spin configuration at different stages and corresponding hysteresis loops.

To explore the origin of the ZFC-EB effect, the evolution of the FM/AFM spin interface based on the simple spin bidomain model was proposed, and the schematic diagram of the spin configuration at different stages is shown in Fig. 4. According to the bidomain model, The FM unmagnetized initial state can be seen as two FM domains with opposite magnetization direction along the direction of the magnetic field and the net magnetization is zero. The FM domain configuration would be imprinted in the AFM during cooling, resulting in the two types of AFM domains with opposite oriented directions at the interface, and both of them align parallel to their neighboring FM domain respectively, as shown in Fig. 4(a). Applying the initial magnetization field will result in FM anisotropy being parallel to the direction of the magnetic field as well as a new fixed interface though the domains growth of FM domains at the expense of AFM domain. It is generally acknowledged that if the interfacial AFM spins are parallel (antiparallel) to the FM spins, the FM (AFM) coupling between them will result in negative (positive) shift of the magnetic hysteresis loop along the magnetic field axis. Hence, two types of interfacial spin configurations after applying the initial magnetization field, corresponding to positive and negative ZFC-EB effect, can be proposed. As shown in Fig. 4(b), under low initial magnetization field, the interfacial AFM spins paralleling to the direction of the magnetic field will merge into FM domains directly, showing AFM coupling and positive shift of the hysteresis loop. While in case of the high initial magnetization field, the high initial magnetization field is large enough to overcome the interfacial AFM anisotropy energy and drives the AFM spins in the new interface formed under low initial magnetization field to rotate from negative direction to the positive direction, and then merge into FM domains, leading to the negative ZFC-EB effect as shown in Fig. 4(c). In another word, low high initial magnetization field is in favor of the positive EB effect while high initial magnetization field is in favor of the negative EB effect. The shift direction of the hysteresis loop can be associated with the competition of the two kind of FM/AFM interfacial spin structure.

Hence, the results in the La_0.5_Sr_0.5_Mn_0.8_Co_0.2_O_3_ ceramics can be associated with the competition of the two ways of forming the AFM unidirectional anisotropy, highlighting the importance of the AFM spins anisotropy on the sign of ZFC-EB effect. Increasing the initial magnetization field firstly would increase the AFM unidirectional anisotropy that parallels to the direction of initial magnetization field, resulting in the strengthening of the AFM coupling at the interface and larger positive shift and *H*_EB_. Further increasing the field, the rotation of the AFM spins from negative direction to the positive direction would reduce the AFM anisotropy as well as *H*_EB_. With the increase of the interface with the AFM anisotropy antiparallel to the direction of initial magnetization field, the enhancement of the negative ZFC-EB effect with the increase of initial magnetization field can be expected. The slight increase of *H*_EB_ (the decrease of |*H*_EB_|) at higher 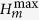 (>110 kOe) may be attributed to the change of bulk AFM spin structure under large applied magnetic field.

**Figure 5:**
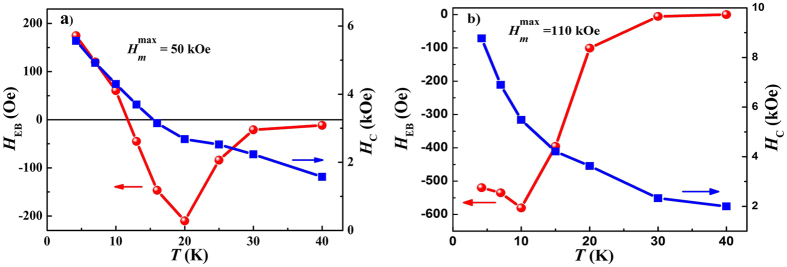
Temperature dependence of *H*_EB_ and *H*_C_ with 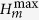 = 50 kOe (**a**) and 110 kOe (**b**) after ZFC.

Usually in the negative FC and ZFC-EB effects, the absolute value of *H*_EB_ (*H*_EB_ < 0) approximately linearly decreases with increasing temperature and gradually disappears around the blocking temperature *T*_B_, which can be attributed to the decrease of the AFM anisotropy paralleling to the direction of positive magnetic field with the increase of temperature^13^,^16^,^17^,^18^,^19^,^20^,^21^,^29^. However, if there is AFM bidomain in the initial state, the decrease of the anisotropy of the right AFM domain would decrease the 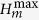 needed to generate the rotation of the interfacial AFM spins from negative to positive direction. Thus, the enhancement of the AFM anisotropy paralleling to the direction of positive magnetic field would be beneficial to the positive ZFC-EB effect at low temperature. The *M*(*H*) loops at different temperatures with 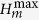 = 50 and 110 kOe were measured after ZFC from 300 K, and the temperature dependence of *H*_EB_ and *H*_C_ are show in Fig. 5. In both cases *H*_C_ decreases with the increase of the temperature. It is worth noting that *H*_EB_ for 50 kOe decreases firstly with the increase of temperature, then changes to negative, reaching a negative peak before approaching to zero at 40 K. The temperature of 40 K can be referred as exchange blocking temperature, *T*_*B*_. A negative peak can also be observed for 110 kOe, indicating the AFM anisotropy parallel to the direction of positive magnetic field at low temperature. All these results are consistent with the discussions above, confirming the reasonable of the model shown in Fig. 4.

## Discussion

Magnetic properties of La_0.5_Sr_0.5_Mn_1−x_Co_x_O_3_ (0 ≤ x ≤ 1) series of samples were studied systematically, and only La_0.5_Sr_0.5_Mn_0.8_Co_0.2_O_3_ exhibits ZFC-EB. As reported before, the formation of new stable FM–SG interface with unidirectional anisotropy is usually though the percolation of isolated FM domains in the initial magnetization process^17^,^21^. Hence, the ZEC-EB effect only occurs in system with appropriate magnetic structure, and the size of initial FM clusters (domains) play a critical role^13^,^16^,^17^,^21^. If the size of initial FM domains is too small, it is hard for the FM clusters to be merged with neighboring FM domains, then no EB effect can be observed. The magnetic state of the parent compound La_0.5_Sr_0.5_MnO_3_ exhibits FM ground state with AFM clusters in low-temperature region^30^,^31^. Upon Co doping, the double exchange interaction between Mn^3+^ and Mn^4+^ would be partially destroyed and some of the FM ground state would be transformed to a robust AFM state^32^,^33^. Then the magnetic state evolves into a coexistence of FM clusters and AFM matrix for x = 0.2 sample, leading to the ZFC-EB effect. However, further increasing the doping level would weaken FM interaction to a higher degree, and the FM clusters in the samples are too small to obtain ZFC-EB effect. Hence, our results are in accordance with the discussions according to the model shown in Fig. 4.

In summary, we have observed positive ZFC-EB effect in La_0.5_Sr_0.5_Mn_0.8_Co_0.2_O_3_ ceramics, and it can transfer into negative ZFC-EB effect with the increase of magnetic field and measuring temperature. Such phenomenon can be attributed to different orientation of interfacial AFM anisotropy formed in the process of initial magnetization. The results here will enrich the type of EB effect, improve the spin bidomain model with variable interface, and benefit the deep understanding of the physical mechanism of exchange bias.

## Methods

The La_0.5_Sr_0.5_Mn_0.8_Co_0.2_O_3_ ceramics was prepared by sintering corresponding powders derived from sol–gel precursors. A stoichiometric amount of La(NO_3_)_3_·6H_2_O, SrCO_3_, Mn(NO_3_)_2_·4H_2_O and Co(NO_3_)_2_·6H_2_O, and appropriate citric acid and nitric acid were used as starting materials. At the end of the process, the gel was first dried at 120 °C and then decomposed at 400 °C to result in dark brown powders, which were ground, then pressed into strip and sintered at 1300 °C for 10 h to produce the finished sample. The good crystallinity of an orthorhombic phase structure without any impurity phases was confirmed by X-ray diffraction (XRD). The magnetic properties were measured by superconducting quantum interference device (SQUID-VSM, Quantum Design) magnetometer and physical property measurement system (PPMS, Quantum Design).

## Additional Information

**How to cite this article**: Shang, C. *et al*. Positive to negative zero-field cooled exchange bias in La_0.5_Sr_0.5_Mn_0.8_Co_0.2_O_3_ ceramics. *Sci. Rep.* **6**, 25703; doi: 10.1038/srep25703 (2016).
